# Reduced-intensity versus Myeloablative Conditioning Regimens for Younger Adults with Acute Myeloid Leukemia and Myelodysplastic Syndrome: A systematic review and meta-analysis

**DOI:** 10.7150/jca.46081

**Published:** 2020-07-06

**Authors:** Shengling Ma, Wei Shi, Ziying Li, Liang Tang, Huafang Wang, Linghui Xia, Yu Hu

**Affiliations:** 1 Institute of Hematology, Union Hospital, Tongji Medical College, Huazhong University of Science and Technology, Wuhan, China.; 2 Fred Hutchinson Cancer Research Center, Seattle, WA 98109-1024, USA.

**Keywords:** myeloablative, stem cell, acute myeloid leukemia

## Abstract

**Background:** Historically, reduced-intensity conditioning (RIC) was recommended to be performed for older patients who were considered ineligible for myeloablative conditioning (MAC) before allogeneic hematopoietic stem cell transplantation (allo-HSCT). However, the evidence regarding the optimal conditioning intensity in younger patients with AML or MDS is weak and contradictory.

**Methods:** PubMed, Medline, Embase, and other online sources were searched from the initial period to February 25, 2020. Odds ratios and 95% confidence intervals were calculated to estimate pooling effects.

**Results:** Four randomized controlled trials (RCTs) about conditioning intensity involving 633 patients were included. There were no significant differences of 1/2/4/5 years progression-free survival (PFS) and relapse incidence (RI) between two conditioning intensities. Overall survival (OS) was similar at 1/2/4 years, but patients receiving RIC had a higher OS at 5 years. Additionally, RIC were associated with lower non-relapse mortality, less grade II-IV and grade III-IV acute graft-versus-host disease (GVHD), and lower incidence of chronic GVHD compared with MAC regimens. Subgroup analysis showed similar OS and RI for AML patients, and there was a trend towards lower NRM and grade II-IV aGVHD in RIC group. Available data for MDS indicated that OS, PFS, and RI were comparable. For intermediate-risk patients, there was no evidence that RIC is inferior to MAC. However, for high-risk patients, MAC tends to perform better.

**Conclusions:** Based on the above results, it might be concluded that RIC is a feasible treatment option for adults with AML or MDS younger than 66 years, particularly those with intermediate-risk disease. Future RCTs incorporating of risk stratifications are warranted to guide the optimal decision under certain conditions.

## Introduction

Allogeneic hematopoietic stem cell transplantation (allo-HSCT) is the backbone therapy for patients with acute myeloid leukemia (AML) or myelodysplastic syndromes (MDS) 1. Historically, reduced-intensity conditioning (RIC) regimens prior to allo-HSCT were typically the strategy in older patients or younger patients with complicated comorbidities that are intolerable of myeloablative conditioning (MAC) regimens. However, ongoing interest has been shown in RIC regimens as consolidation therapy due to lower NRM and comparable survival, which were reported to be performed in two-thirds of transplanted patients in the past two decades 2-4. A series of retrospective studies have drawn contradictory conclusions about the optimum conditioning intensity of younger AML/MDS patients 5-11. The variability in age, performance status, and different comorbidities interfere with the ability to isolate the effects of conditioning intensities on outcomes.

To date, RIC allogeneic HSCT is a feasible treatment option for AML patients aged 60 years or older in the most-updated NCCN clinical practice guidelines (V2.2020) 12 still, for younger patients, there is a lack of high-level evidence for the use of RIC regimens 13. Therefore, we conducted a timely systematic review and meta-analysis of recent randomized controlled trials (RCTs) to provide quantitative evidence on the overall efficacy and toxicity of RIC versus MAC in younger adults with AML or MDS.

## Methods

### Searching strategy

According to the recommendations of the Cochrane Collaboration 14, 15, we retrieved articles from PubMed, Medline, Embase, and Google scholar from their inception until February 25, 2020. Abstracts from the conference proceedings, ongoing and unpublished trials in https://www.clinicaltrials.gov and the bibliographies of other relevant reviews were also manually identified.

We used a combination of corresponding keywords: “allogeneic hematopoietic stem cell transplantation”, “reduced-intensity”, “myeloablative”, “acute myeloid leukemia” and “myelodysplastic syndromes”.

### Definitions of outcomes

The primary outcomes included overall survival (OS) and progression-free survival (PFS). The secondary outcomes consisted of non-relapse mortality (NRM), relapse incidence (RI), incidence of acute graft-versus-host disease (aGVHD), and chronic GVHD (cGVHD). The concept of PFS covered relapse-free survival, disease-free survival and leukemia-free survival. Similarly, the concept of NRM included transplant-related mortality (TRM).

### Selection criteria and study selection

We included all comparative studies that met the following criteria: (1) RCTs included AML/MDS patients in complete remission who received either RIC or MAC; (2) the study reported sufficient patient demographics and outcomes.

To perform a systematic and comprehensive assessment, we combined all the multiple publications. Two investigators (Ma and Shi) selected the studies independently. Any discrepancy regarding eligibility was solved by consulting the senior investigator (Hu).

### Data extraction and quality assessment

Two investigators (Ma, Shi) independently extracted the following information from each eligible study: characteristics (first author, year of the last publication, country, sample size, follow-up time, participant numbers of each group, period of enrollment, recruitment period), participant characteristics(sex, the median age at enrollment, diagnosis), intervention details (conditioning regimens, transplantation details) and outcomes. We contacted all corresponding authors for insufficient data in the articles. We assessed the methodological bias of the included trials by the Cochrane Collaboration's tool 16. The senior author (Hu) assessed all disagreements in bias.

### Data synthesis

Discontinuous data were pooled and calculated as odds ratios (OR) by using a 95% confidence interval (CI). If the publications did not provide data at a particular time point but curves, we used the methods reported by Tierney 17 and Parmer 18.

The summary effect estimates of OR for individual RCTs are indicated by solid squares, with a size proportional to the sample size and the number of events. Horizontal lines indicate 95% confidence interval. The diamonds indicate CIs for pooled effects, with the size of the box relating to the weight of the study. We assessed the heterogeneity among studies by the Q test (P<0.05 to be indicative of statistically significant) and Higgins I^2^ parameters (25-50%, 50-75%, and >75% were divided into low, moderate, and high heterogeneity) 19. As indicated by DerSimonian and Laird 20, if I^2^ ≥ 50%, we chose the random-effect model; otherwise, we used the fixed-effect model. In the sensitivity analysis, the origin of heterogeneity was evaluated by repeating the meta*-*analysis after removing one study at a time. Moreover, we conducted subgroup analyses by diagnoses with the data available in included RCTs.

All statistical analyses were performed using Review Manager 5.3. All tests were 2-sided, and P<0.05 was considered statistically significant.

## Results

### Literature search and study characteristics

**Figure 1:**
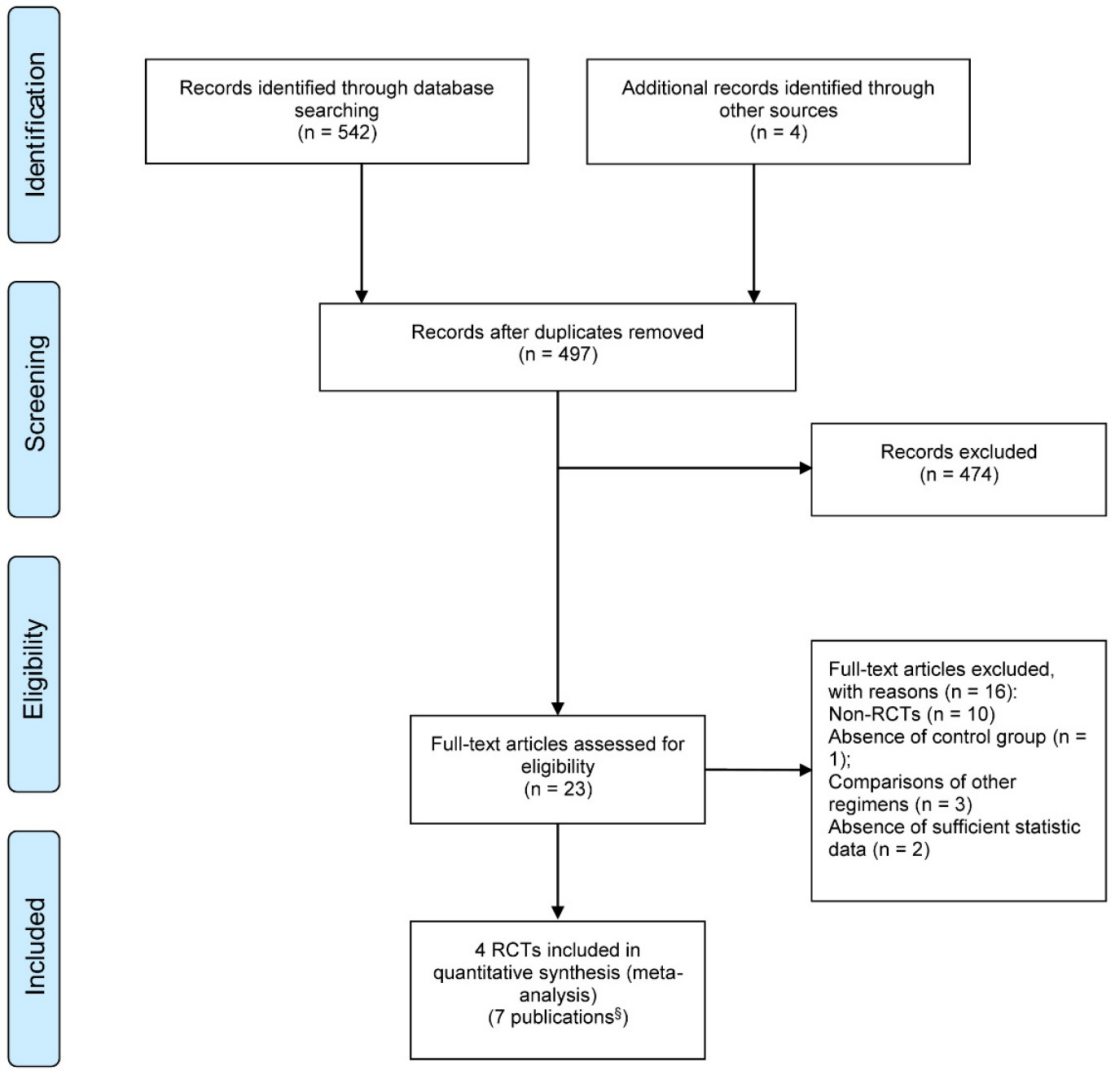
PRISMA flow diagram of study selection; § Includes 4 full text articles and 3 updated publications. RCT: randomized controlled trials.

Figure 1 summarized the processes of study selection. After two rounds of careful screening and hand searches, 4 RCTs 21-24 reported in 7 publications were initially selected for this systematic review, including two abstract proceedings presenting long term follow-ups 25, 26 and an updated report 27 of the previously published one 22, 23. We excluded two non-randomized prospective studies 28, 29.

Table 1 depicts the main characteristics of the included RCTs. Among them, three studies were performed in Europe and one in the United States. All included studies were published from 2013 to 2018. Altogether, 633 patients were randomized to be treated with either RIC (319 [50.4%]) or MAC (314 [49.6%]). Four hundred fifty-four patients were diagnosed as AML, 169 were MDS, and the others were CML or missing. Three RCTs 21-23 applied a uniform conditioning regimen in each arm and a uniform GVHD prophylaxis regimen, whereas several choices were available in the trial of Scott et al. 24. The reported enrolled patients ranged in age from 18 to 66 years, and the median ages of each eligible study varied from 44 to 54.8.

### Risk of bias

**Figure 2:**
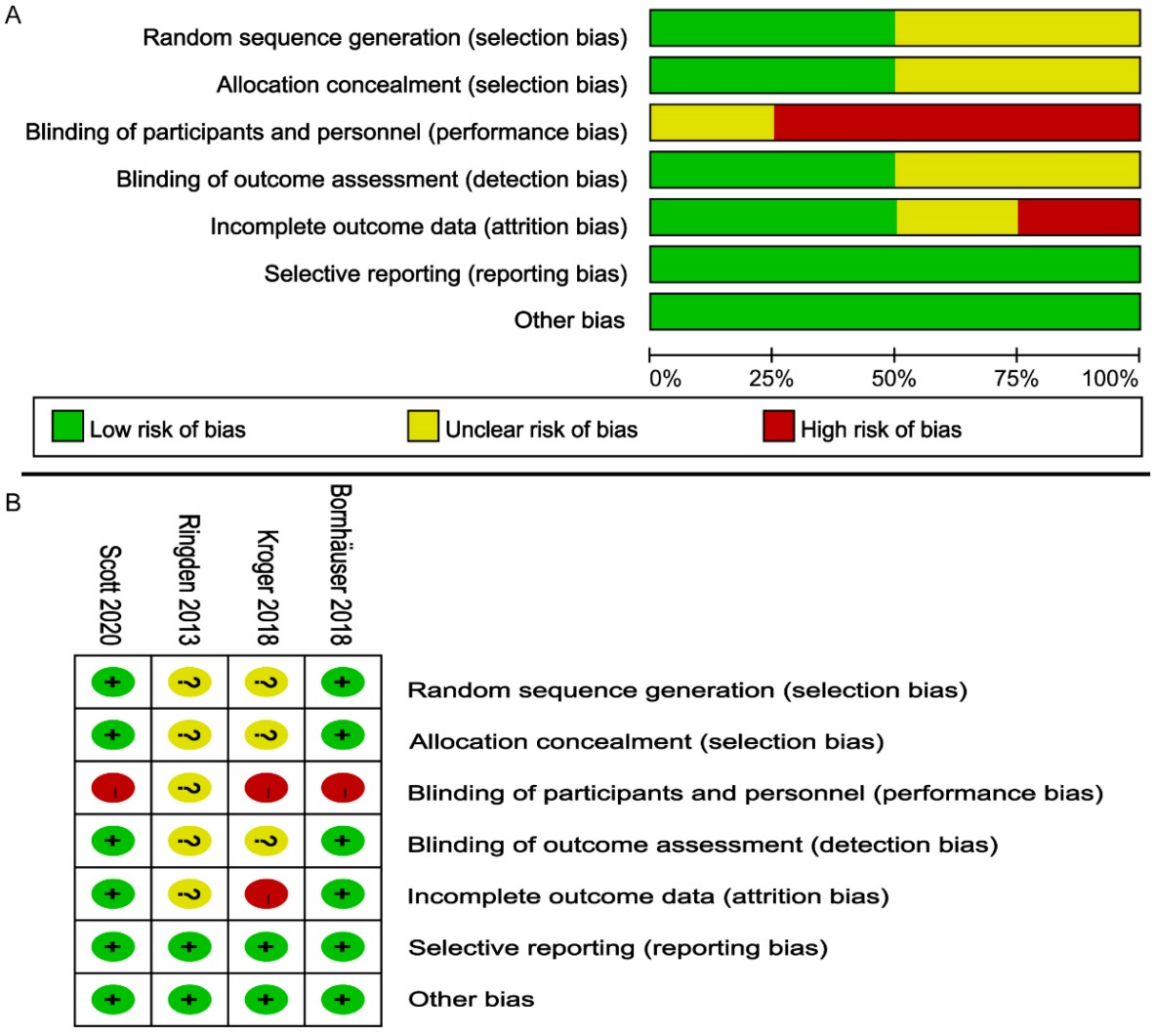
Risk of bias assessment on the included 4 RCTs; (**A**) Risk of bias graph; (**B**) Risk of bias summary.

According to the Cochrane Handbook, the overall bias risk of these RCTs was judged to be low to moderate (Figure 2). All RCTs reported the randomization details. Due to the special nature of transplantation, none of the four studies used double-blind methods. The outcomes of 2 trials 24, 27 were assessed with a low risk of bias, and missing data were described 24, 27. All four studies were free of selective outcomes reporting. Four trials were judged to have a low risk of other bias.

### Effect on overall survival

**Figure 3:**
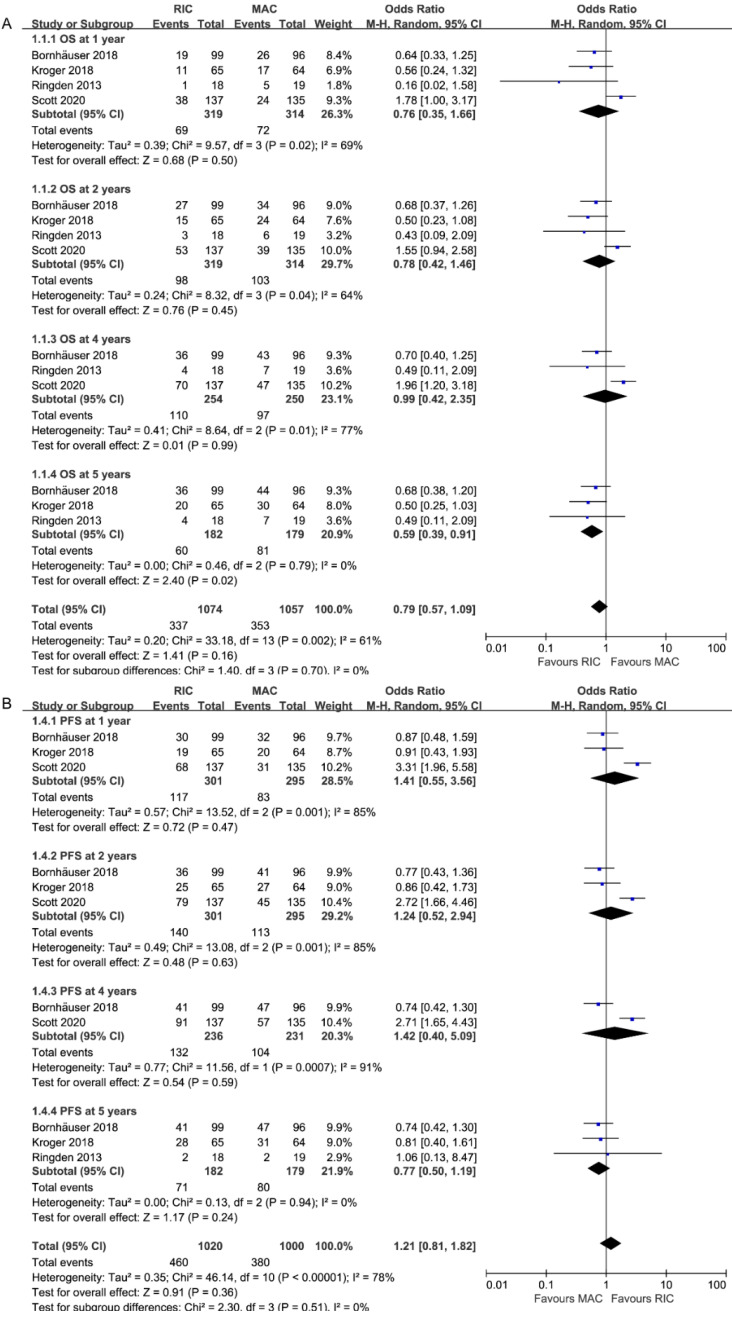
Forest plots for (**A**) Overall survival (OS) and (**B**) Progression-free survival at 1/2/4/5 years.

OS rates were all extractable from the survival curves in 4 RCTs (four for OS at 1 and 2 years, three for 4 26, 27 and 5 years 27, Figure 3A). Specifically, two of them were from the updated abstracts 25, 26. The rates of OS at 1, 2 and 4 years were similar between patients who received RIC and those who received MAC (OR = 0.78, 95% CI 0.27-2.24, P=0.65, I^2^=84%; OR=1.27, 95% CI 0.68-2.38, P=0.45, I^2^=64%; OR=1.01, 95% CI 0.42-2.38, P=0.99, I^2^=77%). However, OS was statistically significantly better with RIC instead of MAC at 5 years (OR=1.69, 95% CI 1.10-2.59, P=0.02, I^2^=0%), in line with the tendency in the second-largest RCT 27 that RIC achieved similar OS (61% at 3 years, 60% at 10 years) but MAC resulted in significantly lower survival at 10 years (47%) compared to that at 3 years (58%). Three trials presented comparable OS at the end of the study except one 26, which reported better OS for MAC at 4 years (65% vs. 49%, p=0.02). Accounting for the moderate and high heterogeneities in subgroups at 2 and 4 years, the I^2^ was valued 0% after repeating the meta*-*analysis removing Scott et al.'s study.

### Effect on progression-free survival

As shown in Figure 3B, PFS data, which comprised a total of 596 patients, were provided at 1, 2, and 4 years, while data from 361 patients were analyzed at 5 years. The pooled ORs were comparable: 0.71 (95% CI 0.28-1.80; P=0.47) at 1 year, 0.81(95% CI 0.34-1.92, P=0.63) at 2 years, 0.83(95% CI 0.35-1.98, P=0.67) at 4 years and 1.30(95% CI 0.85-1.99, P=0.23) at 5 years. Similar to OS, heterogeneity at 1, 2, 4 years (I^2=^85%) disappeared when Scott et al.'s study was ruled out.

### Effect on non-relapse mortality

**Figure 4:**
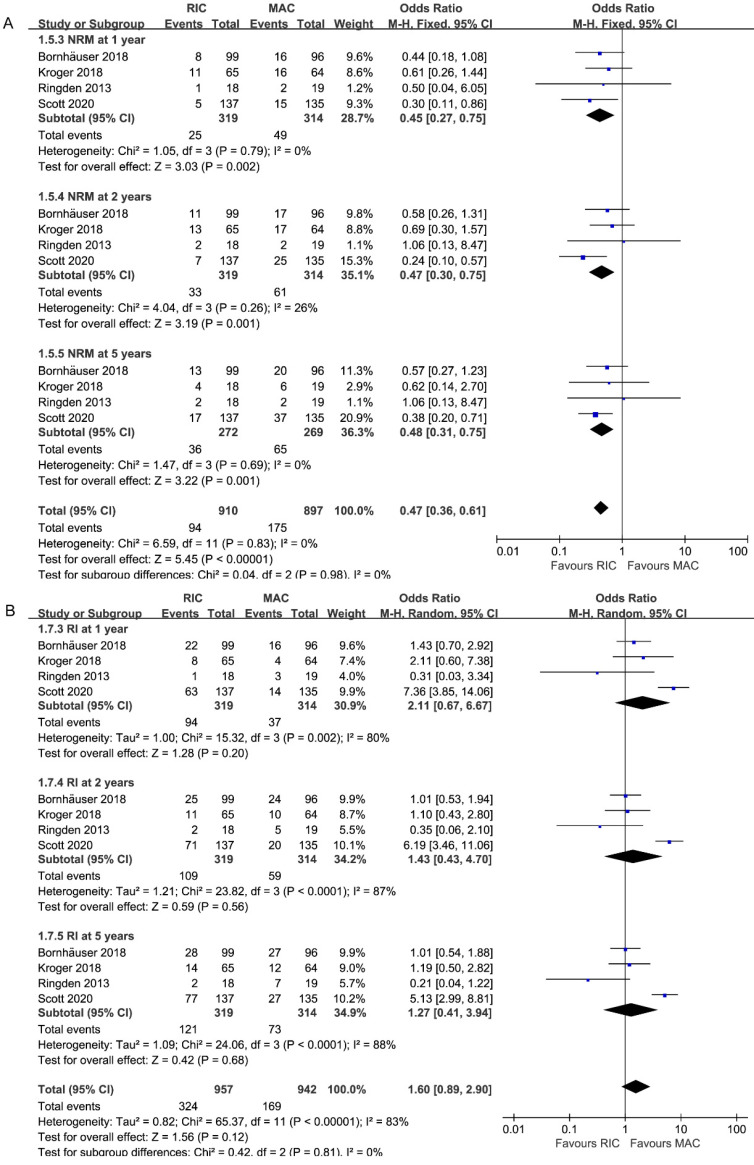
Forest plots for (**A**) non-relapse mortality (NRM) and (**B**) incidence of relapse (RI) at 1/2/5 years.

All studies, including 633 patients, reported data on NRM at 1, 2, 5 years. As depicted in Figure 4A, the combined ORs for 1, 2, 5-year NRM were 0.45 (95% CI 0.27-0.75; P=0.002), 0.47 (95% CI 0.30-0.75; P=0.001) and 0.48 (95% CI 0.31-0.75; P=0.001), respectively. Without heterogeneity in all subgroups (I^2^=0%), additional subgroup or sensitivity analyses were unnecessary. Accordingly, there was strong evidence for the reduced incidence of NRM in the RIC intervention group.

### Effect on relapse incidence

Pooled analysis of all studies presented no statistically difference between two interventions among different follow-up durations (1 year: 2.11, 95% CI 0.67-6.67, P=0.20; 2 years: 1.43, 95% CI 0.43-4.70, P=0.56; 5 years: 1.27, 95% CI 0.41-3.94, P=0.68; Figure 4B). While similar relapse risk was found in the other three trials, only one study 26 reported higher relapse incidence, contributing to the high heterogeneity in three subgroups (I^2^=80%, 87%, 88%).

### Effect on graft-versus-host disease

**Figure 5:**
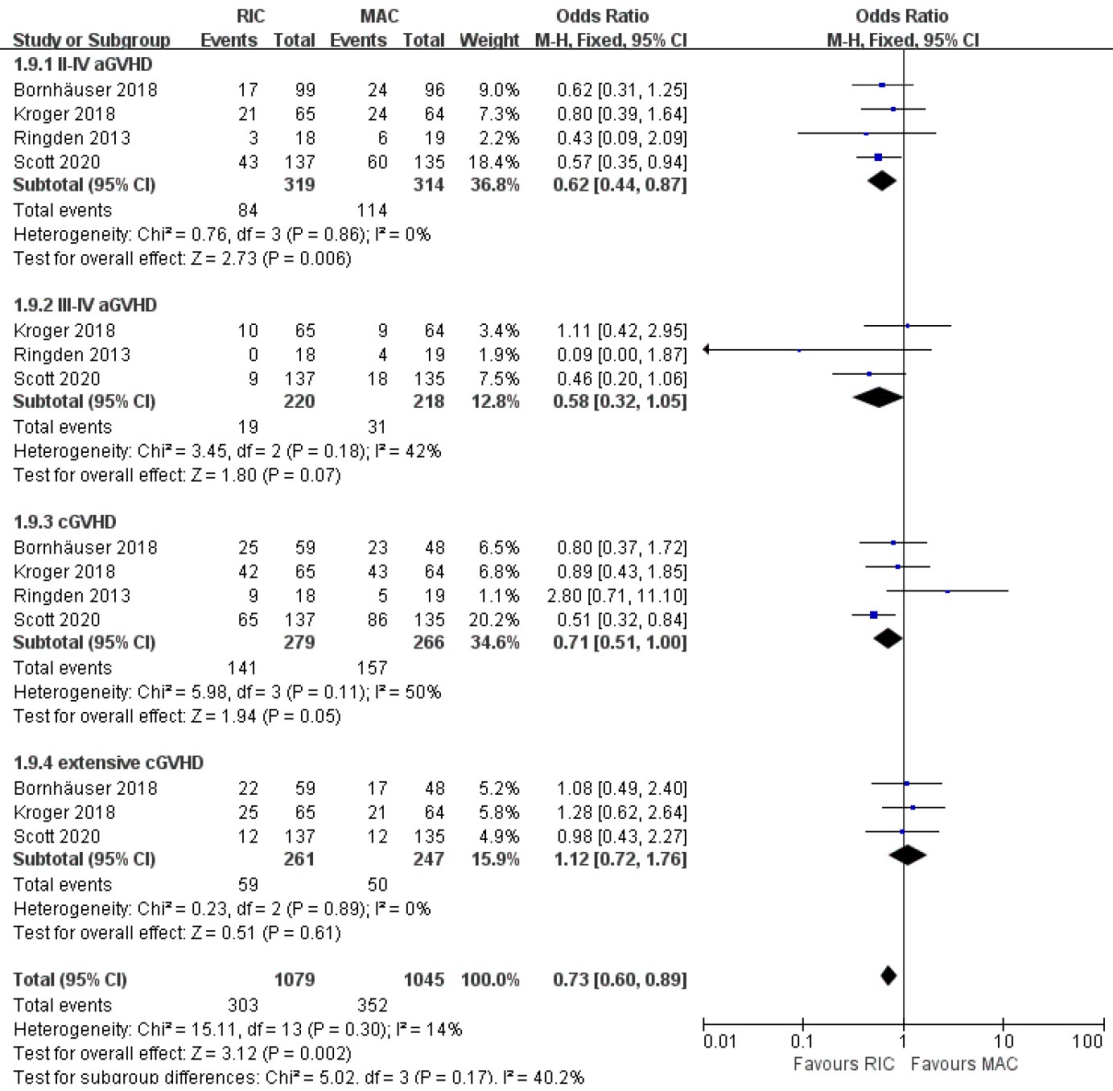
Forest plots for incidence of grade II-IV and grade III-IV acute graft-versus-host disease (aGVHD), overall and extensive chronic graft-versus-host disease (cGVHD) at the end of studies.

As shown in Figure 5, cumulative incidences of acute GVHD grade II to IV and grade III to IV were evaluated in all studies for 633 patients and three of four studies for 438 patients, respectively. The grade II to IV aGVHD were significantly less frequent in the RIC group (OR 0.62; 95% CI 0.44-0.87; P=0.006) compared with the MAC group. There was no statistically significant heterogeneity among the trials (I^2^=0%). Regarding more severe aGVHD (grades III to IV), there was a non-significant trend between the two intensities. (OR 0.58; 95% CI 0.32-1.05; P=0.07) with low heterogeneity among the trials (I^2^=42%). At the end of follow-up, the cumulative incidence of overall and extensive chronic GVHD was obtained in four and three studies, respectively. There was evidence for significantly lower rates of overall cGVHD in the RIC arm (OR 0.71; 95% CI 0.51-1.00, P=0.05) with moderate heterogeneity (I^2^=50%). However, the available evidence from three trials including 508 patients was not sufficiently powered to show a statistically significant difference in extensive cGVHD between the two intervention groups (OR 1.12; 95% CI 0.72-1.76, P=0.61) with extremely low (I^2^=0%) heterogeneity.

### Subgroup analyses focusing on AML

**Figure 6:**
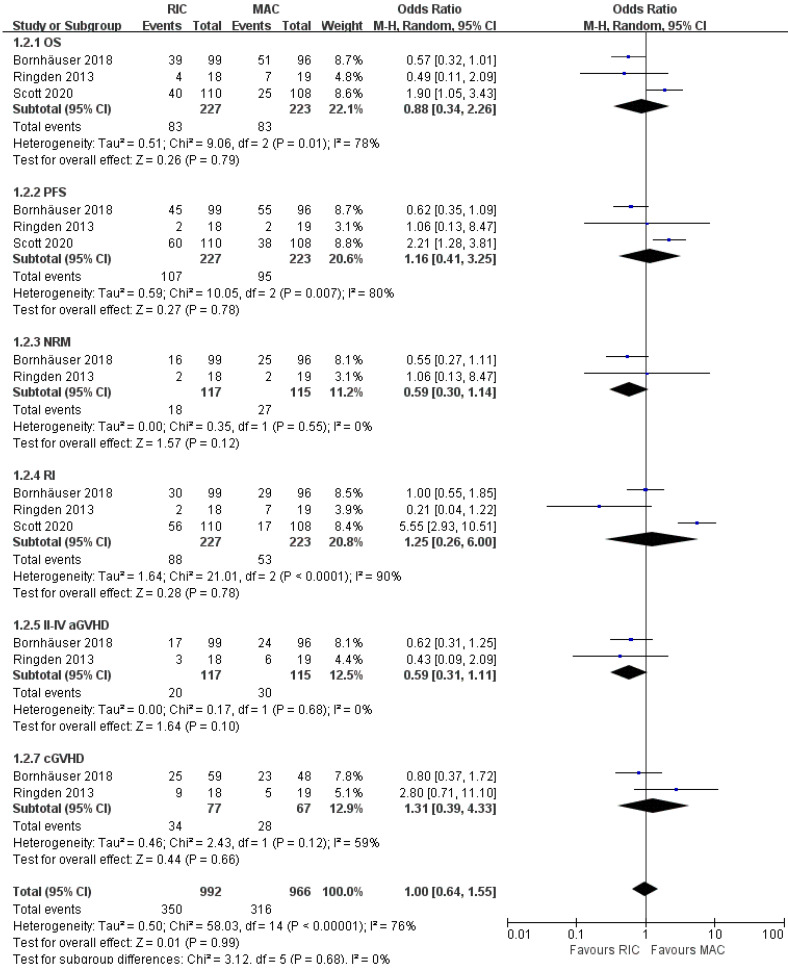
Forest plots for the subset of patients with acute myeloid leukemia. OS, overall survival; PFS, progression-free survival; NRM, non-relapse mortality; RI, relapse incidence; aGVHD, acute graft-versus-host disease; cGVHD, chronic graft-versus-host disease.

Treatment effects were evaluable for 450 patients with AML from three of four trials 21, 23, 24. As presented in Figure 6, OS and PFS were not significantly different between the two intensity regimens, with pooled OR of 0.88 (95% CI 0.34-2.26, P=0.79) and 1.16 (95% CI 0.41-3.25, P=0.78), respectively. Besides, the comprehensive analyses from two trials 21, 23 indicated a potential improvement in NRM and grade II-IV aGVHD for RIC compared with MAC (OR 0.59; 95% CI 0.30-1.14, P=0.12 for NRM, OR 0.59; 95% CI 0.31-1.11, P=0.10 for grade II-IV aGVHD), and the incidences of relapse and overall cGVHD were comparable at the end of the study. Furthermore, in risk stratification by cytogenetics, two studies 23, 24 reported comparable outcomes for intermediate-risk patients, while the smallest RCT 21 indicated better PFS in the RIC group. For high-risk patients with AML, Scott et al. 24 claimed worse OS with RIC, whereas Rinden et al. 21 showed a PFS benefit and Bornhäuser et al. presented no significant difference in OS, PFS, RI, NRM between two arms (Table 2).

### Subgroup analyses focusing on MDS

**Figure 7:**
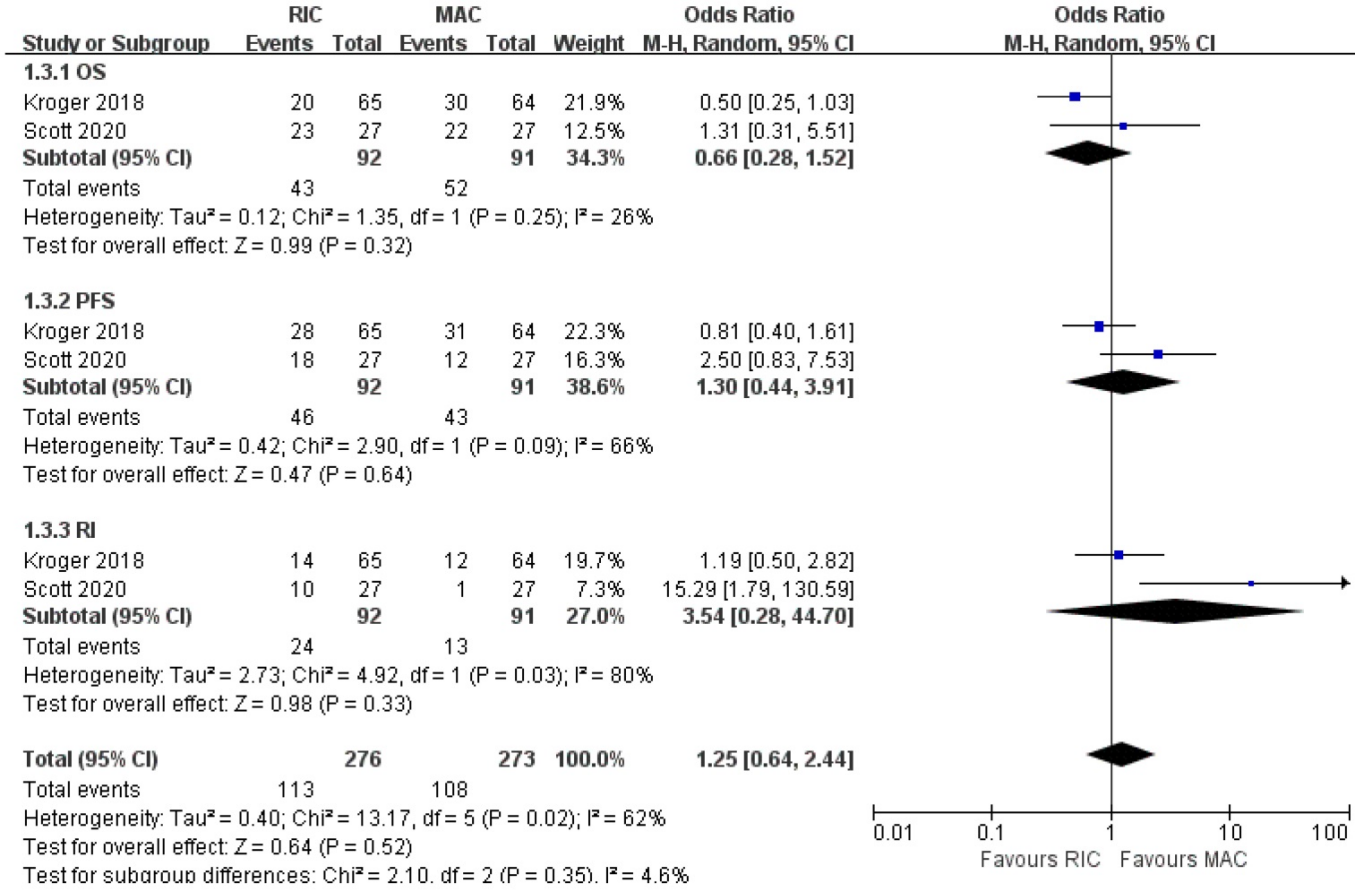
Forest plots for the subset of patients with myelodysplastic syndrome. OS, overall survival; PFS, progression-free survival; RI, relapse incidence.

As shown in Figure 7, two out of four trials involving 183 patients with MDS reported OS, PFS, and relapse incidence. Likewise, none of the pooled results was statistically significant between the two conditioning intensities. For NRM, aGVHD and cGVHD, one study 22 provided similar effects in two arms, whereas the other 24 only reported them for AML and MDS patients as a whole, suggesting better outcomes in RIC regimens. The trial conducted by Kroger et al. 22 stratified cytogenetic risks into three levels and indicated that RIC in the low-risk cytogenetic group resulted in lower NRM but similar NRM in intermediate- and high-risk groups. In the BMT CTN 0901 24, no significant difference was found in OS for standard-risk patients, while RIC in the high-risk cytogenetic group resulted in worse OS (Table 3).

## Discussion

In this systematic review, we included all high-quality RCTs and yielded two main conclusions: Regarding toxicity, we noted that RIC was associated with a lower risk of NRM and GVHD. Besides, the survival and relapse rates of the two regimens were comparable. Although not all studies present five-year data, we have found that the RIC group had a better five-year OS. For subset analyses of AML patients, we found that RIC had similar survival and relapse risk compared with MAC, with a trend towards less NRM and grade II-IV GVHD in RIC regimens. For patients with MDS, available data indicated comparable outcomes in OS, PFS and relapse.

The risk bias of published RCTs was evaluated as low to moderate with randomized settings. Avoiding the selection bias and recalling bias, this meta-analysis provides the highest current level of evidence for the question in hand for AML and MDS patients. Although previous systematic reviews 30-32 have reported the similar results as our meta-analysis except for higher relapse rate, such studies mainly included retrospective studies, in which RIC was generally used for older individuals while MAC for younger patients. Also, the individualized decisions of different attending physicians may inevitably interfere with the conclusions. The advantage of NRM and GVHD reminds us that the RIC regimen is a valuable choice for patients who cannot tolerate high-intensity conditioning 33-35. A current retrospective study revealed that MAC improves outcomes for AML and MDS patients with relatively lower disease risk index (DRI) but has a similar impact on patients with higher DRI 36. Nowadays, more and more novel strategies were being successfully used as subsequent maintenance therapy after allo-HSCT, which significantly reduced the recurrence of allo-HSCT recipients receiving RIC regimens 37-39. Additionally, the long-term GVHD/relapse-free survival was also comparable in two arms for AML patients 40-42 but superior in RIC arm for MDS patients 43.

For AML patients, RIC regimens have demonstrated at least non-inferior survival and a trend of less toxicity. A sizeable observational analysis by the EBMT focusing on 2974 middle-aged (40-60) patients with AML 44 demonstrated that RIC resulted in higher OS and comparable RI in low-risk AML. However, the OS was similar and RI was higher in the intermediate- or high-risk patients. Additionally, the NRM was lower in all three cytogenetic risk groups, which was consistent with our findings in Figure 4A. From another perspective, patients in CR1 with high-risk cytogenetics or with MRD positive in genomics or multi-parameter flow cytometry (MFC) can benefit more from the MAC regimens 45-47.

As for the effect on MDS patients, we took account of the updated reports with longer follow-up 25, 26 and yielded results consistent with the previous studies 22, 24, 48. Furthermore, we systematically reviewed that RIC resulted in less NRM in low-risk groups 22 but better OS in high-risk groups 24 for MDS patients. Besides, the genomics of MDS might have the potential to influence the optimal selection of conditioning intensity. CIBMTR research revealed a higher relapse rate in MDS patients with RAS pathway mutations only after RIC. In contrast, conditioning intensity didn't make a difference in outcomes for TP53-mutated MDS 49.

Of note, only one included clinical trial published by Scott and colleagues 26 reported worse outcomes of RIC. By contrast, the other three RCTs supported RIC for at least similar survival and less toxicity. Though the most weight was assigned to study by Scott BL et al. in the meta-analysis because of its largest sample size, there was still no evidence that reduced-intensity conditioning was inferior to MAC in either AML or MDS. Further sensitivity analysis confirmed the robustness of the results. There are several possible sources of heterogeneity. It was noteworthy that the trial by Scott et al. was the only one that included patients beyond CR2 and applied multiple choices of conditioning regimens and GVHD prophylaxis regimens in each intensity arm. Besides, it could conceivably be hypothesized that the heterogeneity could partially derive from the different distributions of cytogenetic risks and potential MRD in included RCTs.

Although stringent criteria were applied to identify and include studies for our meta-analysis, inherently, like any meta-analysis, there are some limitations in our study. Firstly, although stringent criteria have been applied in our meta-analysis, the baseline characteristics were impractical to be unified among studies. For example, one of the studies 21 has only enrolled 18 and 19 patients in two arms, respectively. The study published by Scott et al. included patients beyond CR2, while others restricted to the first or second CR. Secondly, the limited number of RCTs may have an impact on the statistical power of our results. Finally, regimens of different intensities were not completely uniform in included RCTs, though we tried to decrease the bias through heterogeneity analysis and got reliable conclusions.

Our results indicate that RIC is not inferior to MAC for AML patients with comparable post-transplantation survival, relapse risk, as well as potential advantages in NRM and GVHD. For MDS patients, neither survival nor relapse rate was significantly different. Based on the above results, it might be concluded that RIC is a feasible treatment option for adults with AML or MDS younger than 66 years, particularly those with intermediate-risk disease. Future RCTs incorporating of risk stratifications are warranted to guide the optimal decision under certain conditions.

## Figures and Tables

**Table 1:** Characteristics of studies included in the meta-analysis

		Bornhäuser 2018 23, 27		Kroger 2018 22, 25		Ringden 2013 21		Scott 2020 24, 26	
Country		Germany		Germany		Sweden		USA	
Number of centers		13		18		1		32	
Conditioning regimen		RIC	MAC	RIC	MAC	RIC	MAC	RIC	MAC
Protocol		Flu (150mg/m³) + TBI (8 Gy)	TBI (12Gy) + Cy (120 mg/kg)	Flu (150mg/m³) + Bu (8mg/kg orally or 6.4 mg/kg i.v.)	Bu (16mg/kg orally or 12.8mg/kg i.v.) + Cy (120mg/kg)	Flu (180mg/m³) + Bu (8mg/kg)	Bu (16mg/kg) + Cy (120mg/kg)	Flu (120 to 180mg/m^2^) + Bu (≤8 mg/kg orally or 6.4mg/kg i.v.); FluMel	Flu (120-180mg/m^2^) + Bu (16mg/kg orally or 12.8 mg/kg i.v.); Bu + Cy (120mg/kg); TBI (12-14.2Gy) + Cy.
No. of cases		99	96	65	64	18	19	137	135
Male,%		43 (43%)	47 (49%)	38 (59%)	8 (23%)	12 (63%)	11 (79%)	67 (49%)	76 (56%)
Median age(range),y		44 (18-60)	45 (18-60)	51 (22-63)	50 (19-64)	46 (26-61)	45 (22-58)	54.8 (21.9-65.9)	54.8 (21.9-66)
Diagnosis		AML		MDS: 115; sAML: 12; missing: 2		AML: 29; CML: 8		AML: 218; MDS: 54	
GVHD prophylaxis		CsA /MTX		CsA/MTX		CsA/MTX		CNI/MMF, CNI/MTX, Tac/Siro	
Donor type	MRD	59	58	16	17	7	7	58	57
	MUD	28	24	38	36	11	12	58	66
	Other	12	14	11	11	0	0	21	12
Graft source	PBSC	90	90	59	61	17	16	123	127
	BM	9	6	6	3	1	3	14	8
Recuitmentperiod		2004-2009		2004-2012		NR		2011-2014	
Median follow-up(range), month		119 (102-137)	119 (102-138)	72	75	40.8 (6-104.4)	62.4 (14.4-111.6)	50	50

Abbreviations: RIC, reduced-intensity conditioning; MAC, myeloablative conditioning; Flu, fludarabine; TBI, total-body irradiation; Cy, cyclophosphamide; Bu, busulfan; AML, acute myeloid leukemia; MDS, myelodysplastic syndrome; sAML, secondary acute myeloid leukemia; CML, chronic myeloid leukemia; GVHD, graft-versus-host disease; CsA, cyclosporine A; MTX, methotrexate; MMF, mycophenolate mofetil; CNI, calcineurin inhibitor; MRD, matched related donor; MUD, matched unrelated donor; PBSC, peripheral blood stem cell; BM, bone marrow.

**Table 2:** Disease status, cytogenetic risk stratification and outcomes for acute myeloid leukemia

	Disease status	Risk stratification	RIC	MAC	Outcomes
Bornhäuser 2018	CR1; <5% marrow myeloblasts pre-HSCT	Intermediate§	77	70	No significant difference in OS, PFS, RI, NRM
		High¶	22	26	No significant difference in OS, PFS, RI, NRM
Rinden 2013	CR1 or CR2	Intermediate	11	12	3-year PFS was better in RIC group than in MAC group (90% vs. 75%)
		High	3	3	3-year PFS was better in RIC group than in MAC group (67% vs. 0%)
Scott 2020*	CR; <5% marrow myeloblasts pre-HCT	Intermediate†	71	74	No significant difference in OS
		High‡	61	54	MAC was associated with a significant OS benefit.

RIC, reduced-intensity conditioning; MAC, myeloablative conditioning; CR, complete remission; OS, overall survival; PFS, progression-free survival; RI, relapse incidence; NRM, non-relapse mortality. §Intermediate=normal and non-high risk, including normal karyotype and other intermediate abnormalities. ¶Including the following cytogenetic abnormalities: +8; Complex (≥3 aberrations); -5, -7, del(5q); Inv(3), t(3;3); t(6;11), t(11;19); t(6;9). †Defined according to the Eastern Cooperative Oncology Group/SWOG cytogenetic classification schema. ‡Including unfavorable risk cytogenetics according to the Eastern Cooperative Oncology Group/SWOG, FLT3 mutation regardless of accompanied cytogenetic risk. *Data are for patients with acute myeloid leukemia and myelodysplastic syndromes combined.

**Table 3:** Disease status, cytogenetic risk stratification and outcomes for myelodysplastic syndrome

	Disease status	Risk stratification	RIC	MAC	Outcomes
Kroger 2018	MDS: 115; sAML: 12; missing: 2; <20% marrow myeloblasts pre-HCT	Low	28	24	RIC resulted in significantly lower NRM
		Intermediate	13	17	No significant difference in NRM
		High	18	17	No significant difference in NRM
Scott 2020*	<5% marrow myeloblasts pre-HCT	Intermediate	71	74	No significant difference in OS
		High‡	61	54	MAC was associated with a significant OS benefit.

RIC, reduced-intensity conditioning; MAC, myeloablative conditioning; CR, complete remission; MDS, myelodysplastic syndrome; sAML, secondary acute myeloid leukemia; OS, overall survival; NRM, non-relapse mortality. ‡intermediate-II or high-risk disease per the International Prognostic Scoring System. *Data are for patients with acute myeloid leukemia and myelodysplastic syndromes combined.
